# Thermosensitive hydrogel coupled with sodium ascorbyl phosphate promotes human umbilical cord-derived mesenchymal stem cell-mediated skin wound healing in mice

**DOI:** 10.1038/s41598-023-38666-w

**Published:** 2023-07-24

**Authors:** Liji Liu, Sheng Yao, Xianhua Mao, Zheng Fang, Cheng Yang, Yan Zhang

**Affiliations:** 1 Department of Bone and Joint, The Central Hospital of Yueyang, Yueyang, 414020 China; 2 Huarong County People’s Hospital, Yueyang, 414207 China; 3 Yueyang Vocational and Technical College, Yueyang, 414000 China

**Keywords:** Biological techniques, Cell biology, Stem cells

## Abstract

Poor survival and restricted function of transplanted stem cells are regarded as limiting their efficacy in wound recovery greatly. Consequently, it is necessary to identify innovative therapeutic strategies to solve these issues. Firstly, the biological effect of PF-127 hydrogel alone and in combination with SAP on the survival, and migration of cultured HUCMSCs was assessed by cell viability, apoptosis, and scratch wound assays. *S. aureus* and *E. coli* were used to evaluate the antibacterial activity of PF-127 plus SAP combination. Further, the ability of HUCMSCs-conditioned medium (HUCMSCs-CM) to promote the angiogenesis and migration of human umbilical vein endothelial cells (HUVECs) in vitro was evaluated using tube formation and transwell migration assays. Finally, the HUCMSCs embedded in PF-127 plus SAP scaffold were administered onto mice’s excisional cutaneous wound bed. Histological and immunohistochemical analyses were employed to investigate the wound healing capacity as well as cellular responses of PF-127/HUCMSCs/SAP hydrogel. PF-127 showed cytotoxicity on HUCMSCs, whereas the addition of SAP significantly promoted cell viability and alleviated apoptosis of HUCMSCs encapsulated in PF-127 hydrogel in vitro. SAP supplementation substantially abrogated the inhibiting effect of PF-127 on the migration of HUCMSCs in vitro. The combination of PF-127 and SAP exerted an obvious bacteriostatic function on *S. aureus* and *E. coli.* Moreover, the co-treatment with SAP could remarkably enhance the stimulative effect of HUCMSCs-CM on the angiogenesis and migration of HUVECs in vitro. PF-127 combined SAP-embedded HUCMSCs transplantation resulted in a potently accelerated wound healing process, promoted the number of proliferating cells and newly formed blood vessels, as well as enhanced expression of vascular endothelial growth factor. PF-127 coupled with SAP contributes to HUCMSCs-mediated traumatic wound closure in mice by promoting cell survival, antibacterial action, and angiogenesis. Our results offered a theoretical foundation for the clinical treatment of traumatic skin defects.

## Introduction

Traumatic skin injury can bring about the destruction of structural and functional integrity of normal skin tissue^1^. Superficial skin injuries that only damage the epidermis can be thoroughly self-healing, while deep skin wounds damaging the underlying dermis exhibit restricted self-repair capacity, severe microbial infection, and abnormal fibrotic scars^2^. Treating severe skin lesions, such as large cutaneous wounds, serious skin injuries, and skin burns, often requires debridement surgery, supplementing growth factors, or negative pressure therapy with wound bioactive dressings^3,4^. Nonetheless, the therapeutic efficacy of these treatments often seems unsatisfactory for many patients.

Nowadays, mesenchymal stem cells (MSCs)-based therapy has been recognized as an effective approach to tissue regeneration and wound healing via recruiting epithelium, promoting angiogenesis, and regulating inflammation^5,6^. In particular, the Human Umbilical Cord-Derived MSCs (HUCMSCs) are among the most widely used in preclinical and clinical trials due to their harvested repetitiveness, minor invasive collection process, and higher yield of MSCs. HUCMSCs are considered ideal cell sources of regeneration medicine due to their longer longevity, higher proliferative ability, and stronger cell differentiation capacity than other MSCs^7–9^. In previous investigations on wound regeneration, the common method of delivering MSCs is the injection. At the same time, the therapeutic efficiency was limited by poor engraftment, short retention, and low survival of the transplanted MSCs^10–12^.

Due to the recent advances in tissue engineering research, the combination of MSCs with biomaterial-based scaffolds that extend the survival time of MSCs in the wound microenvironment has served as a prospective alternative for rejuvenating wounds. Pluronic F-127 is a heat-sensitive hydrogel that exists as a liquid at low temperatures and as a semisolid gel when the temperature rises^13^. The degradation of PF-127 is rapid after being transplanted in vivo with almost no side effects on the host. The US FDA has authorized PF-127 for the clinical utilization of stem cells^14^. Wharton's jelly MSCs encapsulated in PF-127 hydrogel can significantly promote diabetic wound healing in mice^15^. Wen et al. used PF-127 to deliver the MSCs to spinal fusion in rabbits and reported that the compound was superior to MSCs alone^16^. Another study reported that PF-127 was beneficial for retaining stromal cell-derived extracellular vesicles, and the application of PF-127 improved the repair and regeneration of articular esophageal fistula^17^. However, there are obstacles to applying PF-127 to treat cutaneous wounds on account of it has been reported that PF-127 has a cytotoxic effect on the survival of encapsulated cells^15,18,19^. A study on reducing the cytotoxicity of PF-127 demonstrated that supplying a membrane-stabilizing agent can markedly promote MSCs viability in PF-127^20^.

As a sodium salt of ascorbic acid 2-phosphate, sodium ascorbyl phosphate (SAP) can maintain high stability even when exposed to reactive oxygen species and aqueous solution for a long time^21^. It was found that SAP exerted multiple pharmacological activities, such as antioxidant, anti-inflammatory, immunostimulant, and anti-bacterial actions^22^. The antioxidant property of SAP has been investigated profoundly of late^23^. SAP has also been shown to possess enhanced antioxidative and anti-inflammatory capacities against various disease models when combined with hyaluronic acid biopolymer^24^. Additionally, supplementation of SAP greatly enhances the viability of Wharton’s jelly MSCs embedded in PF-127 and accelerates diabetic wound healing in mice^15^. Nevertheless, studies exploring the effects of SAP on HUCMSCs encapsulated in PF-127 hydrogel are scarce, and the underlying molecular mechanism remains largely unknown.

This research aimed to determine the influence of SAP on the survival of HUCMSCs in PF-127 encapsulation. The therapeutic benefits of the PF-127/HUCMSCs/SAP hydrogel on the skin wound model were evaluated in the present study, and its underlying molecular mechanisms were also investigated.

## Materials and methods

### Cell culture

The HUCMSC line Saliai-HMSC (UC)-N was acquired from Guangzhou Saliai Stem Cell Co., Ltd. The HUCMSC cell line was cultured in DMEM/F12 medium (Life Technologies, Carlsbad, CA) and grown in a humidified incubator with 5% CO_2_ while fed with 10% FBS (Gibco, USA). The HUVECs were obtained from the Bank of Chinese Academy of Sciences. HUVECs were cultured in a MEM-alpha medium (Gibco, USA). For the experiment, cells in the logarithmic phase were utilized.

### Animal

8-week-old C57BL/6 male mice (25 ~ 30 g) were purchased from Hunan Slake Jingda Laboratory Animal Co., Ltd (Changsha, China) and group-housed under the stable temperature (18~22 °C) as well as humidity (50~60%). All animal experiments were approved by the Ethics Committee of The Central Hospital of Yueyang for the use of animals and conducted in accordance with the National Institutes of Health Laboratory Animal Care and Use Guidelines. The animal experiment complies with the ARRIVE guidelines and in accordance with the National Institutes of Health guide for the care and use of Laboratory animals.

### Preparation and characterization of HUCMSCs/PF-127/SAP hydrogel

20% PF-127 hydrogel preparation and stem cell encapsulation was conducted in accordance with the previous report^15,19^. In brief, the PF-127 powder (Sigma, USA) was dissolved in precooled DMEM-F12 medium to make a 20% (w/v) PF-127 solution. The PF-127 solution was clarified with syringe filters (0.2 μm) and stored under 4 °C conditions. HUCMSCs were encapsulated within 20% PF-127 solution with 400 μM or 800 μM of SAP (Sigma, USA) to form the PF-127/ HUCMSCs/SAP hydrogel, and the hybrid was placed in a 37 °C incubator for 15 min to make gel transformation.

The flow characteristics of temperature-sensitive PF-127/ HUCMSCs/SAP hydrogel were observed and photographed at 20 and 37 °C, respectively. Rheological properties of PF-127/ HUCMSCs/SAP hydrogel were measured using a rotary rheometer (Kinexus, Malvern) at a strain amplitude of 1% and a frequency of 0.159 Hz. A Zeiss scanning electron microscope (SEM) was used to observe the microstructural morphology of PF-127 hydrogel and HUCMSCs/PF-127/SAP hydrogel.

### Production of HUCMSCs-conditioned medium (HUCMSCs-CM)

HUCMSCs (1 × 10^6^) were cultured in 6-well plates overnight, and the medium was replaced by serum-free DMEM/F12 medium with 2 mM L-glutamine (Gibco). The HUCMSCs-CM was collected 24 h later and clarified using a 0.22 μm filter. The HUCMSCs-CM was kept in a − 80 °C refrigerator.

### Cell viability assay

To measure cell viability, 1 × 10^4^ HUCMSCs with different encapsulation conditions were cultivated in 96-well plates overnight, and 10 µL of CCK-8 reagent (Beyotime, Beijing) was added to each well and incubated at 37 °C for half an hour. The absorbance at 450 nm was then tested by a microplate reader.

### Cell apoptosis assay

The cell apoptosis was determined in HUCMSCs using the DAPI staining method (BioVision, USA). 5 × 10^4^ HUCMSCs with different encapsulation conditions were cultivated in a 48-well plate for 24 h. HUCMSCs were fixed and permeabilized, after which it was dyed with DAPI for 10 min. The results were then observed and captured using an inverted fluorescence microscope (DCM8, Leica, Germany). Randomly select 20 fields of view, calculate the proportion of apoptotic cells, and calculate the average value.

### Cell wound scratch assay

1 × 10^5^ HUCMSCs with different encapsulation conditions were cultivated in 6-well plates and incubated in an FBS-free medium for 24 h. Then, a 10 μL pipette tip was employed to scratch a straight line on the cell monolayer. HUCMSCs were imaged at the indicated time point, and cell migration rate was calculated by Image J software. Data has been reported as the extent of wound closure by the initial scratch width.

### Tube formation assay

HUVECs were incubated in serum-free MEM-alpha medium with or without HUCMSCs-CM for 12 h. 100 μL of Matrigel (BD Biosciences) was diluted with MEM-alpha medium and coated in 6-well plates at 37 °C for 1 h. Then, 5 × 10^4^ HUVECs were seeded on Matrigel. The 6-well plates were transferred back to the incubator overnight. The tube formation ability of HUVECs was photographed and quantified under phase-contrast inverted microscopy.

### Transwell migration assay

2.5 × 10^4^ HUVECs were incubated in 500 μl serum-free MEM-alpha medium and added to the upper chamber in a Transwell 24-well plates (Corning, USA). The lower chamber was pre-coated with PF127 with or without SAP, and 750 μl complete medium with or without HUCMSCs-CM was placed in the lower chamber. After 12 h of incubation, the lower surface of HUVECs were stained with crystal violet for 15 min. Finally, the migrated HUVECs were photographed and counted with a microscope.

### In vitro antimicrobial activity assay

100 μL hydrogel solution was added to a 24-well plate. 1 mL *S. aureus and E. coli* suspension (1 × 10^4^ CFUs/mL) was supplied to the 24-well plate coated with hydrogel and incubated for another 12 h. Finally, 100 μL of the above bacterial solution was applied to LB agar plates and placed at 37 °C for 24 h. The colony forming unit (CFU) on the plates was counted and calculated according to the previous literature^25^.

### Skin wound model establishment

The full-thickness skin wound modeling in mice was conducted as previously reported^12^. In brief, the mice were anesthetized and placed prone on the operating table. After successively shaving off the hair in the back area with electric scissors and depilatory wax, a full-thickness round wound with a diameter of 12 mm was made with a biopsy punch on the back of each mouse. All the mice were blindly randomized into four groups (n = 6): (1) control group (50 μL DMEM-F12 medium), (2) PF-127 group (50 μL 20% PF-127 solution), (3) PF-127/HUCMSCs group (1 × 10^6^ HUCMSCs were embedded in 50 μL 20% PF-127 solution), (4) PF-127/HUCMSCs/SAP group (1 × 10^6^ HUCMSCs and 400 μM SAP were encapsulated in 50 μL 20% PF-127 solution). All drugs were locally applied to the wound site once daily. The wound site was observed and photographed on days 0, 3, 6, 9, 12, and 15 after surgery. The mice were euthanized 15 days after surgery, then the wound bed area and surrounding samples were immediately dissected for further analysis.

### Histological analysis

Resected tissues were fixed in 4% paraformaldehyde for 15 min, then embedded in paraffin, cut into thin sections, and stained with HE and Masson Staining Kit, respectively. The sections were observed under a light microscope (Olympus, Japan).

### Immunohistochemical analysis

Skin tissues were immobilized in 4% formaldehyde, dehydrated, embedded, and cut into sections. After repairs with 10 mM sodium citrate buffer (pH 6.0, Beyotime) at 94 °C for 15 min, sections were cooled to room temperature and sealed with 1% bovine serum albumin (BSA, Beyotime) for 30 min. Subsequently, sections were treated with primary antibodies targeted to Ki- 67, CD31, and VEGF (Abcam). Next, sections were hatched with secondary antibodies labeled with biotinylation, and re-stained with hematoxylin. The stained sections were eventually captured under a light microscope (Olympus, Tokyo, Japan).

### Statistical analysis

The statistical tests were carried out via GraphPad Prism 6.0. The differences between two or more groups were compared via the one-way ANOVA followed by Tukey’s posttest. *P* less than 0.05 was considered statistically significant.

### Ethics approval

All animal experiments were approved by the Ethics Committee of The Central Hospital of Yueyang for the use of animals and conducted in accordance with the National Institutes of Health Laboratory Animal Care and Use Guidelines. The animal experiment complies with the ARRIVE guidelines and in accordance with the National Institutes of Health guide for the care and use of Laboratory animals.

## Results

### Characterization of hydrogel

**Figure 1:**
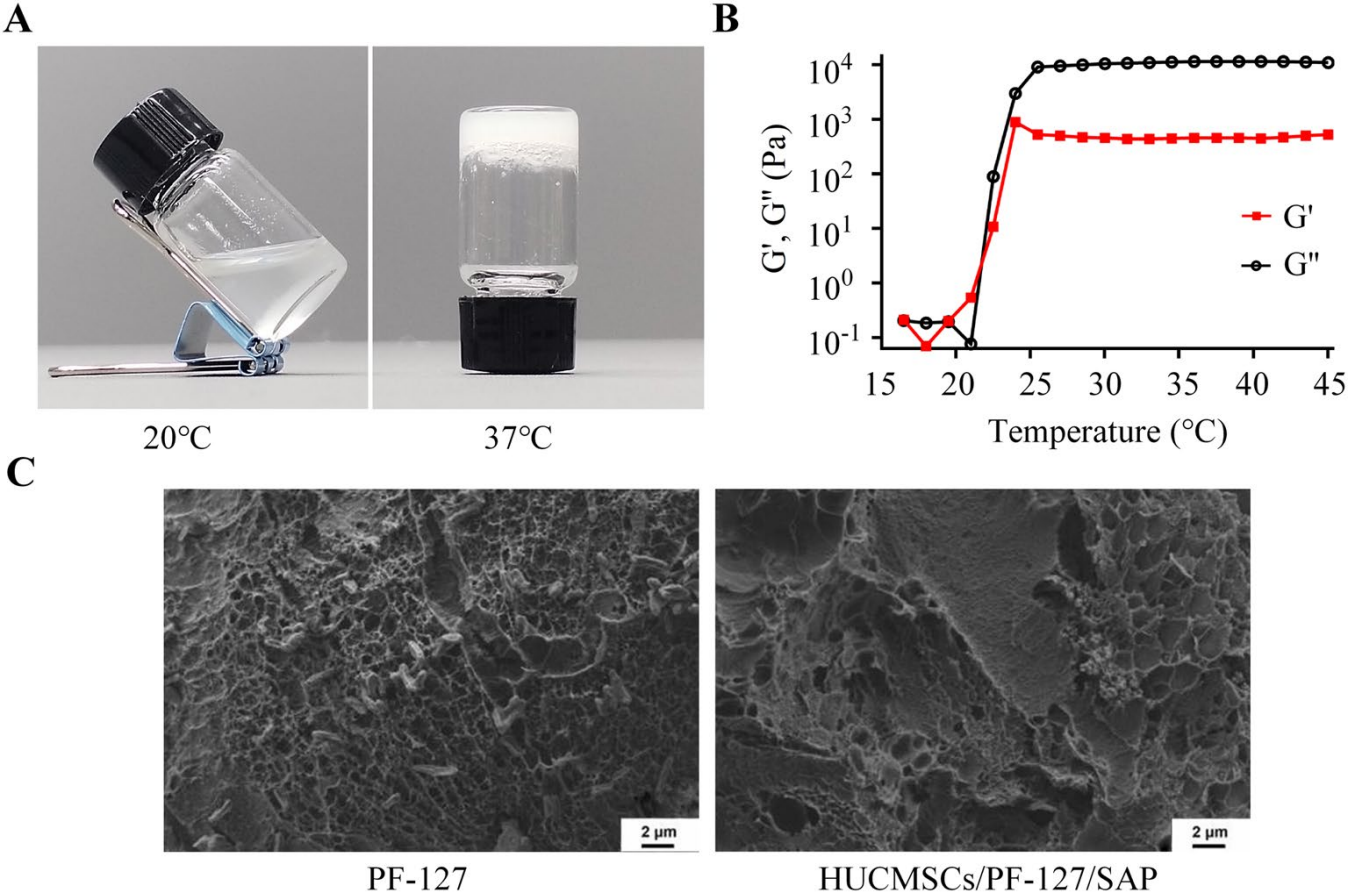
Characterization of PF-127/HUCMSCs/SAP hydrogel. (**A**) Optical photos of the hydrogel at different temperatures. (**B**) Rheological properties of F-127/HUCMSCs/SAP hydrogel from 15 to 45 °C. (**C**) SEM images demonstrating the porous structure of hydrogel.

As shown in Fig. 1A, the PF-127/HUCMSCs/SAP hydrogel is liquid and almost transparent at low temperatures. With the temperature rising gradually (20 ~ 37 °C), the thermosensitive hydrogel transforms into a semisolid gel with a sharp increase in absorbance. In addition, we explored the physical characteristics of PF-127/HUCMSCs/SAP hydrogel by rheological measurements of elastic modulus (G′) and viscous modulus (G″) with increasing temperature from 15 °C to 45 °C. The results showed that the initial gelation temperature of PF-127/HUCMSCs/SAP hydrogel was about 20 °C (Fig. 1B). The morphological features of hydrogels were observed by SEM after lyophilization (Fig. 1C). Both PF127 hydrogel and PF-127/HUCMSCs/SAP hydrogel presented interconnected and similar porous microstructures. The loose and porous network structure exhibits the good absorptivity and breathability of PF-127/HUCMSCs/SAP hydrogel, which facilitates skin wound healing.

### SAP improves the survival and migration of HUCMSCs encapsulated in PF-127 hydrogel

**Figure 2:**
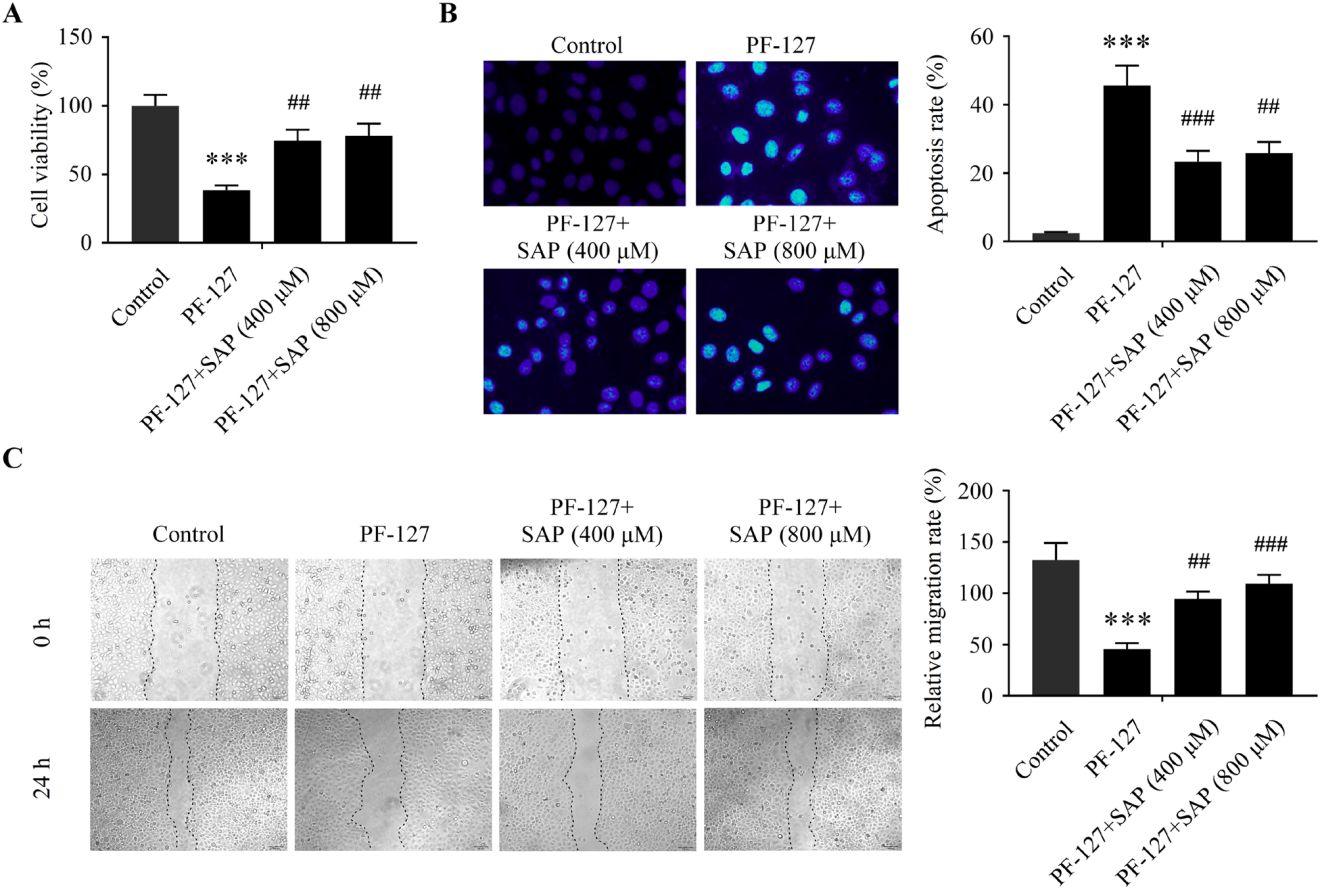
SAP improves HUCMSCs survival and migration in PF-127 encapsulation. (**A**) HUCMSCs were treated with PF-127 or combined with SAP, then the cell viability was determined by CCK-8 assay. n = 6 independent samples. (**B**) Apoptosis of HUCMSCs after being treated with PF-127 or combined with SAP was detected by DAPI staining. n = 20 independent samples. (**C**) HUCMSCs were incubated with PF-127 or combined with SAP, and the migration of HUCMSCs was determined by cell wound scratch assay. n = 3 independent samples. ***P < 0.001 versus control group. ^##^*P* < 0.01, ^###^*P* < 0.001 versus PF-127 group. All data are presented as mean ± SD. Error bars represent SD.

To determine the biological role of SAP on HUCMSCs encapsulated in PF-127 hydrogel. HUCMSCs were cocultured in PF-127 hydrogel with or without SAP for 24 h, and cell viability was determined by CCK-8 assay. As shown in Fig. 2A, the cell viability was markedly reduced after HUCMSCs were encapsulated in PF-127 hydrogel. The co-treatment with SAP (400 μM) could remarkably counterbalance the suppressive effect of PF-127 on the cell viability of HUCMSCs.

We further explored whether apoptosis contributed to the HUCMSCs death caused by PF-127 (Fig. 2B). In accordance with the results of the CCK-8 assay, results of the DAPI staining assay showed the percentage of DAPI-positive PF-127-encapsulated HUCMSCs was significantly higher than that of the control cells. It is noted that the promoting effect of PF-127 on the apoptosis of HUCMSCs was attenuated by the supplementation of SAP (400 μM). Whereas there was no statistical difference in the regulation of cell viability and apoptosis as the concentration of SAP was raised to 800 μM. These discoveries showed that the addition of SAP markedly improves HUCMSCs viability but attenuates the apoptosis of HUCMSCs in PF-127 encapsulation.

We then explored whether PF-127 combined SAP promoted the migration of HUCMSCs by cell wound scratch assay. As anticipated, the migration of HUCMSCs was markedly inhibited by PF-127 (Fig. 2C). Meanwhile, we found that the migration of HUCMSCs was improved significantly following 400 μM SAP or 800 μM SAP applications as compared with the PF-127 group.

### PF-127 plus SAP combination exerts potent an antibacterial effect

**Figure 3:**
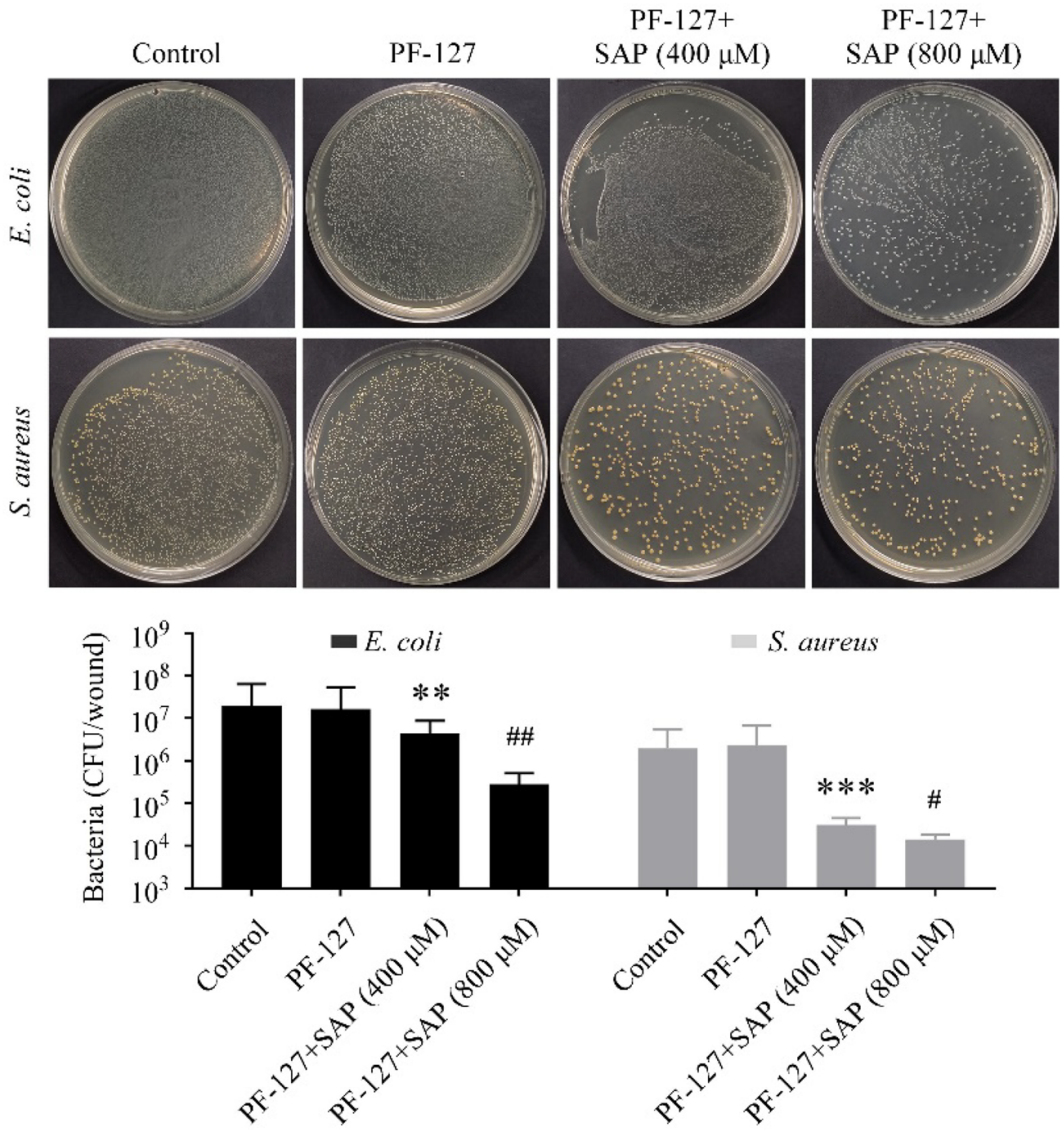
Images of *E. coli* and *S. aureus* incubated with PF-127 hydrogel with different concentrations of SAP. **P < 0.01, ***P < 0.001 versus PF-127 group. ^#^*P* < 0.05, ^##^*P* < 0.01 versus PF-127/SAP (400 μM) group. n = 3 independent samples. Data are presented as mean ± SD. Error bars represent SD.

As shown in Fig. 3, PF-127 exerts almost no inhibitory effect on the growth of *E. coli* and *S. aureus*. The PF-127/SAP hydrogel exerted a strong bacteriostatic function; the colony number in PF-127/SAP (800μM) group was the least abundant compared with other groups. Collectively, the combination of PF-127 and SAP demonstrated powerful antibacterial properties against both Gram-positive and Gram-negative bacteria. Thus, these findings suggest that PF-127/SAP hydrogel could improve HUCMSCs survival as well as reduce the risk of wound infection.

### PF-127 plus SAP combination contributes to HUCMSCs-mediated angiogenesis in vitro

**Figure 4:**
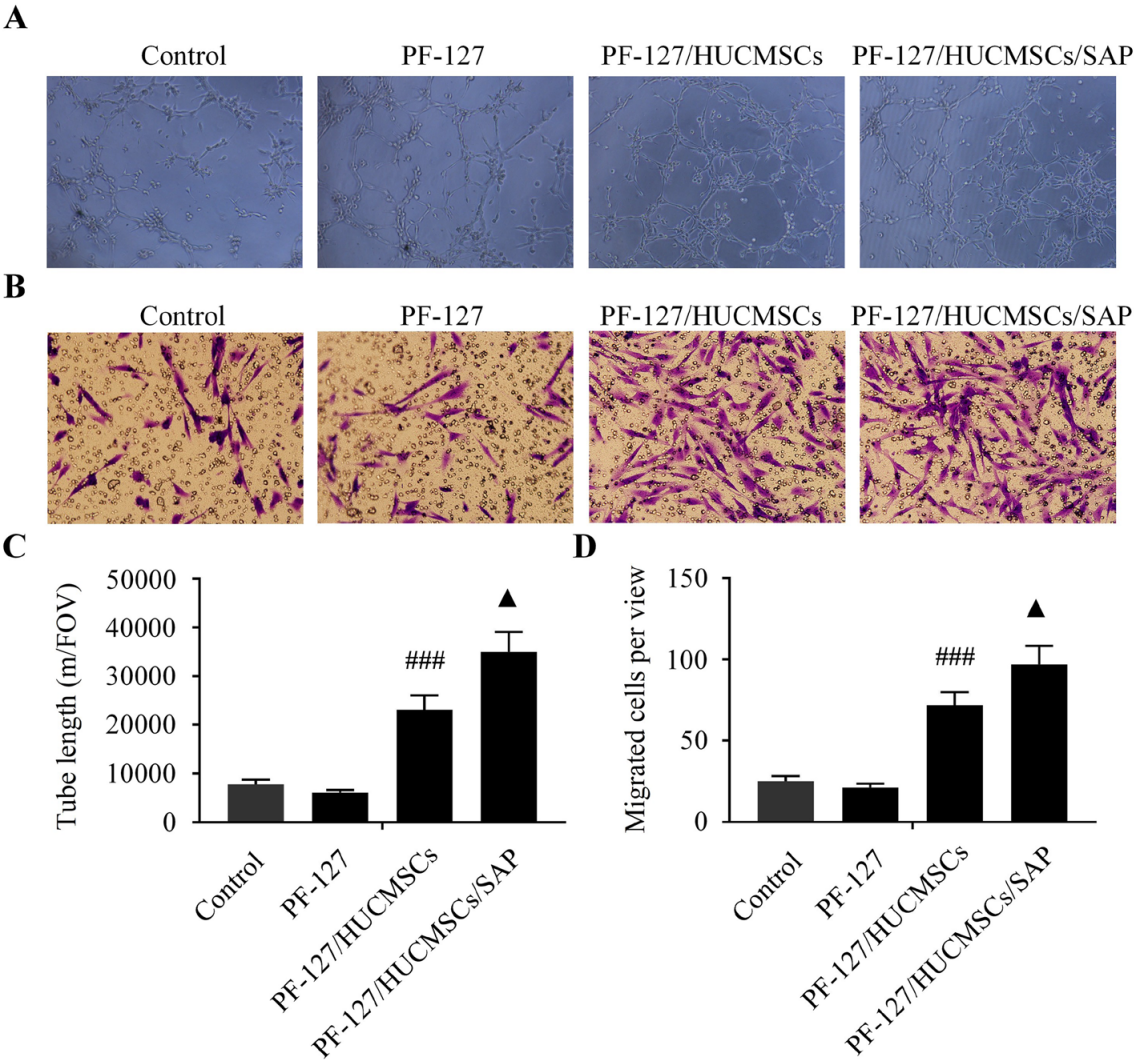
HUCMSCs-CM plus SAP combination restores the dysfunctions of HUVECs in vitro. (**A**) HUVECs were treated with or without HUCMSCs-CM in different encapsulation conditions, then the tube network was determined by tube formation assay. n = 6 independent samples. (**B**) The migration of HUVECs after being treated with or without HUCMSCs-CM in different encapsulation conditions was detected by transwell migration assay. n = 6 independent samples. (**C**) Quantitative analysis of the total tube length in the tube formation assay. (**D**) Quantitative analysis of the migrated cells in the transwell migration assay. ^###^*P* < 0.001 versus PF-127 group. ^▲^*P* < 0.05 versus PF-127/HUCMSCs group. Data are presented as mean ± SD. Error bars represent SD.

Subsequently, tube formation and transwell migration assays were employed to investigate the biological characteristics of HUVECs cultured with or without HUCMSCs-CM in different encapsulation conditions. The tube formation and migration of HUVECs were not obviously Influenced after incubating with 20% PF-127 solution (Fig. 4A,B). HUVECs cultured in PF-127/HUCMSCs-CM formed more tubules per field of view (FOV) than those in the PF-127 group. The number of tubules derived from HUVECs was further promoted by coculturing with PF-127/HUCMSCs/SAP-CM compared to that in the PF-127/HUCMSCs group (Fig. 4C).

Similarly, the transwell migration assay showed that compared with the PF-127 group, PF-127/HUCMSCs-CM significantly promoted the number of migrated HUVECs, and the addition of SAP further enhanced the PF-127/HUCMSCs-CM-induced HUVECs migration (Fig. 4D). These findings showed that SAP could further enhance the restoration of HUVECs function induced by HUCMSCs-CM.

### PF-127 plus SAP combination facilitates HUCMSCs-mediated skin wound healing in vivo

**Figure 5:**
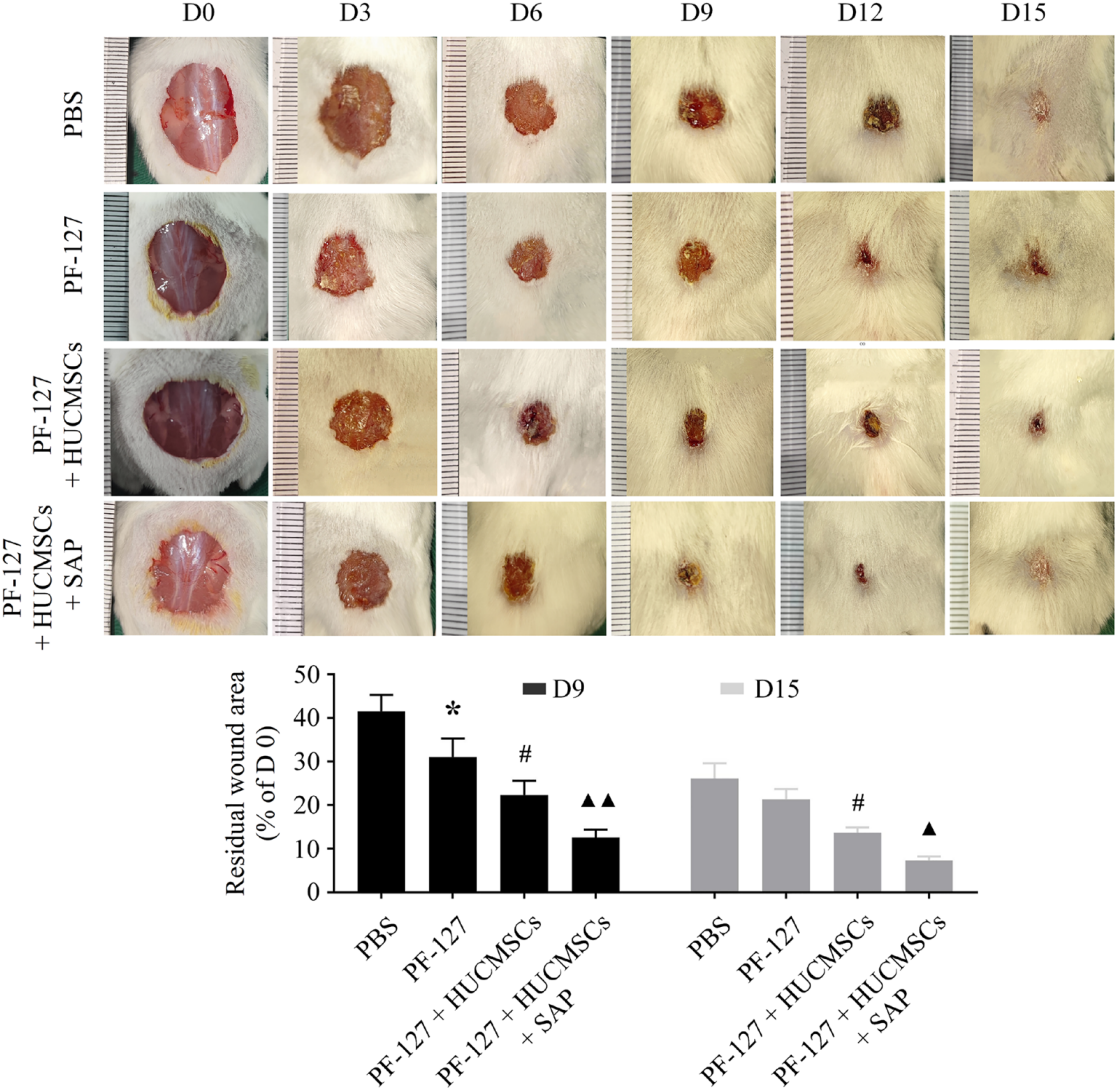
Representative images of the wound healing process in four groups at the indicated time point. ^*^P < 0.05 versus PBS group. ^#^*P* < 0.05 versus PF-127 group. ^▲^*P* < 0.05, ^▲▲^*P* < 0.01 versus PF-127/HUCMSCs group. n = 6 independent samples. Data are presented as mean ± SD. Error bars represent SD.

The above experiments have confirmed that PF-127 coupled with SAP facilitates HUCMSCs-mediated wound closure in vitro. Further study was done to explore the hypothesis that PF-127 plus SAP could contribute to HUCMSCs-mediated wound healing using the full-thickness skin wound model. Similar to our prediction, compared with the control group, remarkable accelerating effects of skin wound recovery have been observed in the PF-127 hydrogel group. The application of PF-127/HUCMSCs led to a further decline in residual wound area at day 9 post-surgery compared to the PF-127 group (Fig. 5). The wound in the PF-127/HUCMSCs/SAP group healed better on day 9 after transplantation than in the other groups. At 15 days after surgery, the skin wounds in the PF-127/HUCMSCs/SAP group almost entirely healed, while other groups still had varying degrees of visible unhealed wounds.

### PF-127 coupled with SAP promotes HUCMSCs-mediated dermis regeneration and collagen deposition

**Figure 6:**
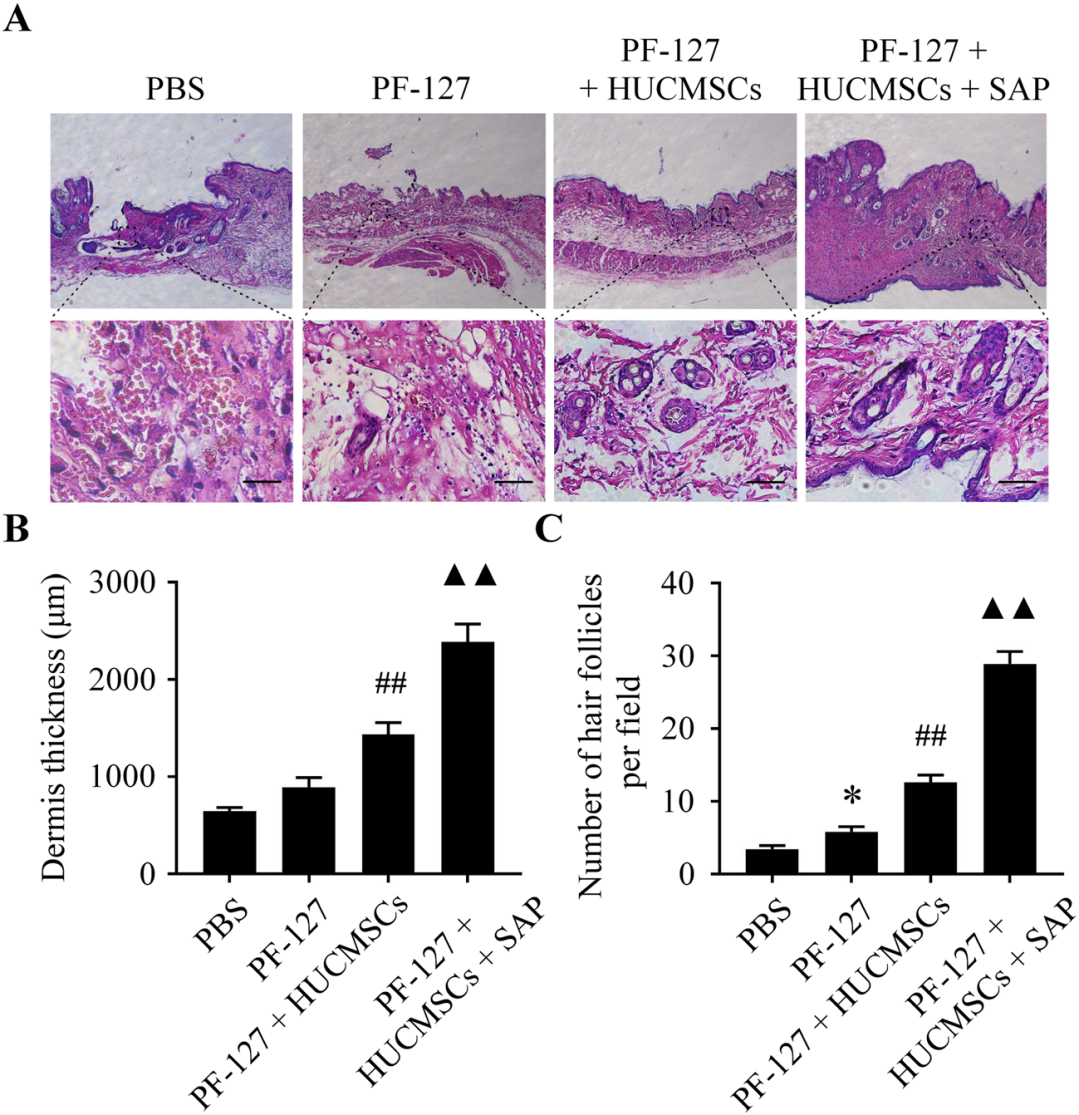
PF-127 coupled with SAP improves HUCMSCs-mediated dermis regeneration in mice. (**A**) Light microscopic pictures of H&E staining of the wound bed and surrounding normal tissue. (**B**) Quantitative analysis of the healed dermis thickness in four groups. (**C**) Quantitative analysis of the number of newborn hair follicles in four groups. *P < 0.05 versus PBS group. ^##^*P* < 0.01 versus PF-127 group. ^▲▲^*P* < 0.01 versus PF-127/HUCMSCs group. n = 6 independent samples. Data are presented as mean ± SD. Error bars represent SD. Scale bar 100 μm.

Histological analysis was conducted to explore further the effect of the PF-127/HUCMSCs/SAP hydrogel on wound healing. As seen in Fig. 6A, at 15 days after surgery, the inflammatory response can be observed in all four groups, while the most serious inflammatory response was seen in the PBS group, which is characterized by local hemorrhagic focus, many inflammatory cells but few collagen fibers, a substantial decline in connective tissue. In the group of PF-127/HUCMSCs, the inflammation was lessened or subsided. In sharp contrast, tissues in the PF-127/HUCMSCs/SAP group showed the least inflammatory cells. The dermis thickness in the PF-127/HUCMSCs/SAP group was thicker than other groups (Fig. 6B), along with a relatively intact epithelium layer and abundant fibroblasts in the dermis. H&E staining also illustrated that the PF-127/HUCMSCs/SAP group exhibited a smaller residual wound area than other groups at day 15 post-surgery. The statistical data also verified that the quantity of newborn hair follicles was markedly higher in all scaffold groups when compared with that in the control group (Fig. 6C), and the PF-127/HUCMSCs/SAP group still showed the highest amount of hair follicles.

**Figure 7:**
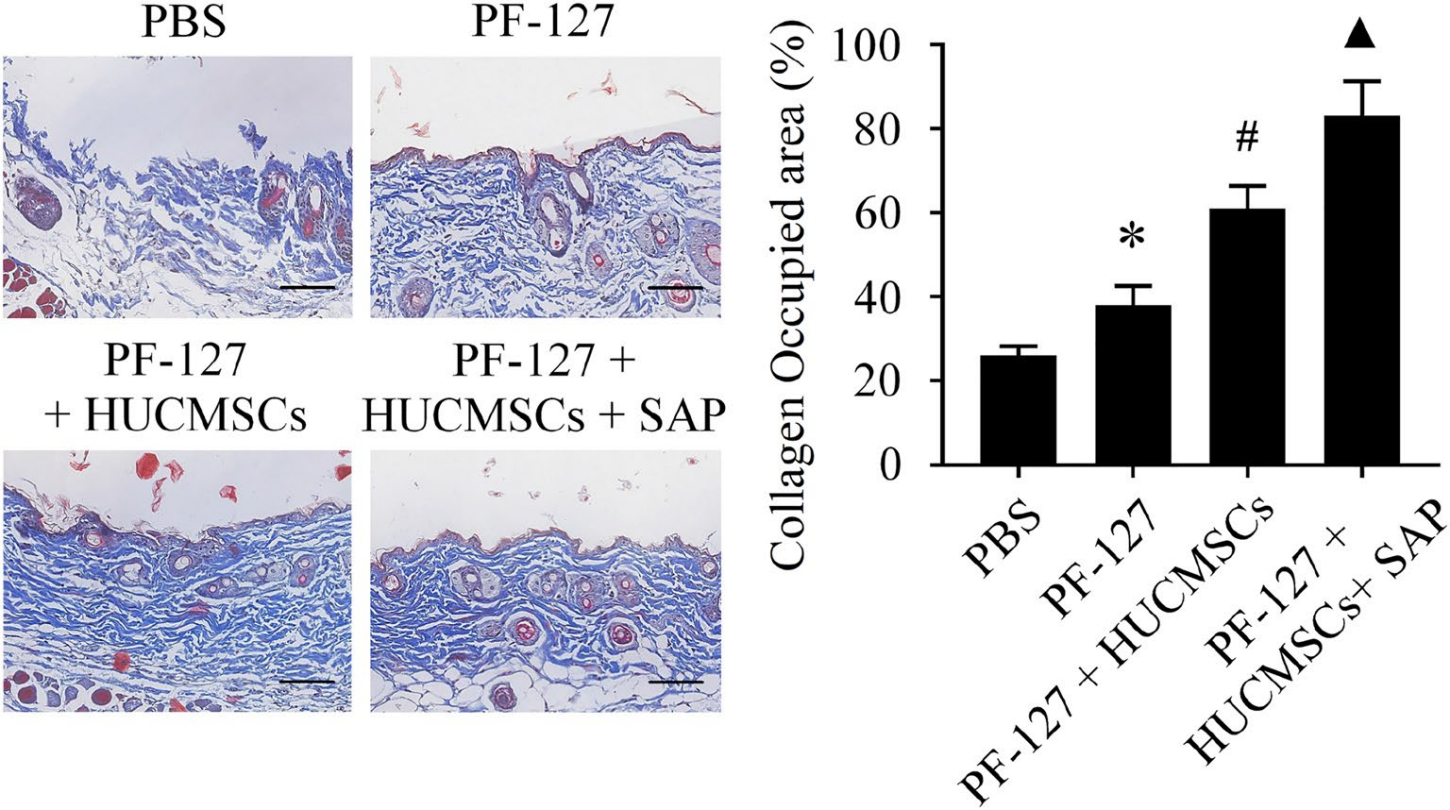
Light microscopic pictures of Masson’s trichrome staining of the wound bed and surrounding normal tissues. *P < 0.05 versus PBS group. ^#^*P* < 0.05 versus PF-127 group. ^▲^*P* < 0.05 versus PF-127/HUCMSCs group. n = 6 independent samples. Data are presented as mean ± SD. Error bars represent SD. Scale bar 200 μm.

Additionally, the collagen fibers disposition in the wound area tissues was determined by Masson staining. The present study showed that the proportion of collagen fibers deposited at the regenerated dermis area in the PF-127/HUCMSCs/SAP group is much higher than in other groups (Fig. 7).

### PF-127 combined with SAP improves HUCMSCs-mediated cell proliferation and angiogenesis in vivo

**Figure 8:**
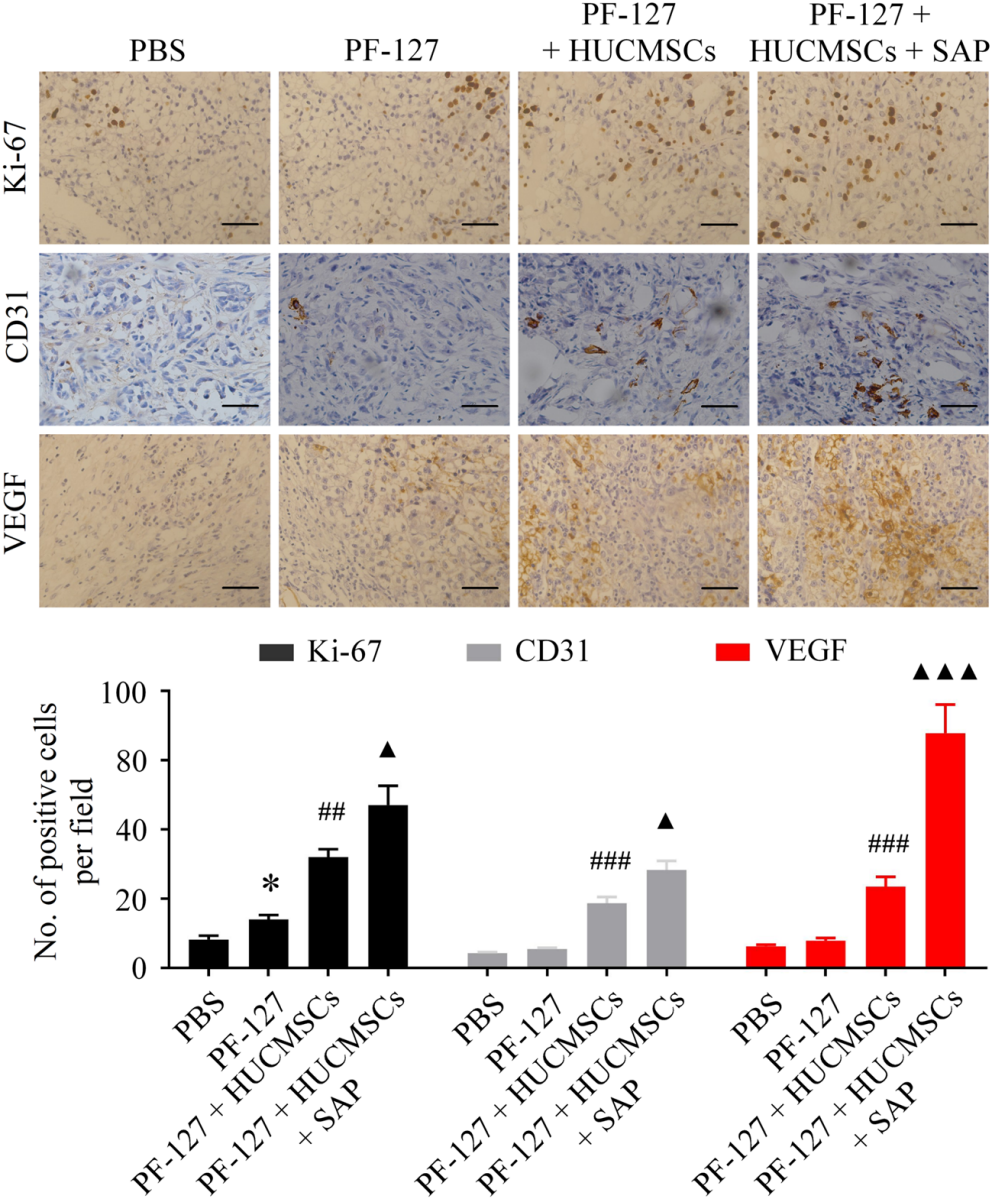
Light microscopic pictures of immunohistochemical staining of Ki-67, CD31, and VEGF in the wound bed and surrounding normal tissues. *P < 0.05 versus PBS group. ^##^*P* < 0.01, ^###^*P* < 0.001 versus PF-127 group. ^▲^*P* < 0.05, ^▲▲▲^*P* < 0.001 versus PF-127/HUCMSCs group. n = 6 independent samples. Data are presented as mean ± SD. Error bars represent SD. Scale bar 100 μm.

We further explore the mechanism underlying PF-127 plus SAP combination improves HUCMSCs-mediated wound healing. Ki-67, CD31 and, VEGF, which are related to proliferation and angiogenesis, are evaluated by immunohistochemical staining. As shown in Fig. 8, the Ki-67 positive cells in the tissues of the PF-127/HUCMSCs/SAP group were significantly higher compared to other groups. Our study showed that the number of newly formed blood vessels in the PF-127/HUCMSCs/SAP group was significantly increased than the other groups. Furthermore, the expression of VEGF was conducted to evaluate angiogenesis. VEGF-positive cells could be found in all four groups, while its positive cell number was highest in the PF-127/HUCMSCs/SAP group. Therefore, these results revealed that PF-127 plus SAP combination improves HUCMSCs-mediated wound healing by promoting cellular proliferation and angiogenesis.

## Discussion

An ideal wound dressing material is not restricted to preventing external infections, but also to guiding skin cell infiltration. Adequate vapor permeability, easily removable, and good flexibility are all essential requirements for wound dressing^26,27^. Among these various wound dressings, polymeric hydrogels offer many advantages, such as good hydrophilicity, excellent biocompatibility, and adjustable physical properties, which are identified as one of the most promising wound dressing material candidates^13^. As one of the most commonly used polymeric hydrogels, the porous structure of PF-127 acts as a solid barrier against external infections, provides moisturization and absorbs extrudates^14^. In the present study, the local application of PF-127 hydrogel alone could promote skin wound healing, but the effect could be more satisfactory, similar to previous reports^15,18,19^. Recently, a tissue engineering-based strategy has shown that the application of biomaterial-based scaffolds coupled with stem cells, drugs, or biologically active factors in the regeneration of skin wounds has gained more popularity and attention^10–13^. In the present study, 20% PF-127 served as a delivery system to load HUCMSCs/SAP complexes, and the therapeutic effect of the PF-127/HUCMSCs/SAP hydrogel on full-thickness cutaneous wound healing was investigated.

Many basic and clinical studies have proven that HUCMSCs are a promising drug candidate for wound healing in human and animal skin defects due to their beneficial properties, such as the stimulation of angiogenesis, the regulation of immunity, and the promotion of cell regeneration^7–9^. However, due to stem cell suspension run-off after topical injection at the transplanted site, less than 1% of transplanted stem cells remain at the injection site for more than a week. So, the therapeutic efficacy of stem cells was markedly attenuated by poor cell engraftment. In order to solve this issue, embedding cells in biomaterial-based scaffolds to confine injected cells at the location of transplantation without run-off has been deeply assessed^15–17^. IVFK hydrogels provide cell attachment sites for mouse myoblasts, significantly prolonging cell survival and supporting their expansion and differentiation into myotube myocytes^28^. It was reported that the combination of engineered fibrin hydrogel and MSCs could localize more cells at the wound site, which revealed better wound repair effects than the transplantation of MSCs alone^29^. Another study demonstrated that stem cells encapsulated in chitosan hydrogel sustained a high cell density for a long time, and this combination significantly promoted wound healing^30^. The thermo-sensitive feature of PF-127 hydrogel facilitates cell attachment and retention at the transplantation location, which prompted PF-127 to be widely used in the field of tissue regeneration^14^. In our study, PF-127/HUCMSCs hydrogel could be adequately filled in irregular skin wounds evenly in a solution condition. After transplanting onto the skin defects, the fluidity of PF-127/HUCMSCs hydrogel gradually decreased with the temperature increase. The hydrogel becomes condensed eventually and separates the skin defect from the external environment to reduce the possibility of wound infection. The application of PF-127/HUCMSCs remarkably speeds up the repair of skin wounds compared to the PF-127 group. At the same time, PF-127 can upregulate the expression level of intracellular ROS, resulting in cytotoxic effects on encapsulated cells^15,18,19^. In line with these findings, results from the current study consistently uncovered that the survival and migration of encapsulated HUCMSCs were significantly attenuated after incubation in 20% PF-127 hydrogel for 24 h. Therefore, poor cell survival and biological characteristics of MSCs in PF-127 encapsulation were other obstacles that limited the therapeutic efficacy of stem cells.

Researchers have made a lot of attempts to improve the therapeutic effect of transplanted stem cells. A recent study has shown that red OLED light can enhance the angiogenesis, cell adhesion, and migration capabilities of human adipose-derived mesenchymal stem cells^31^. Many studies have focused on the addition of cell membrane-stabilizing agents to the PF-127 hydrogel to improve cell survival. Hydrocortisone was generally added to the PF-127 gel to improve cell viability, such as HepG2 and HMEC-1 cells^32^. However, hydrocortisone has an immunosuppressive effect on the body, which adversely affecting wound recovery. Meanwhile, the application of natural product vitamin C can increase the viability of PF-127-encapsulated BMSCs^32^. Vitamin C has a powerful antioxidant effect, and vitamin C alone can effectively inhibit bacterial growth and promote wound recovery, so it is an ideal cell membrane stabilizer^33^. As one kind of vitamin C analog, SAP is characterized by higher physical-chemical stability than ascorbic acid^21^. This study also observed that SAP can effectively inhibit bacterial growth in vitro. Furthermore, supplementation of SAP attenuates the cytotoxic effect of PF-127 and enhances cell survival in rat MSCs encapsulation^15^. Given that SAP has a strong ROS scavenging ability, we hypothesize that adding SAP to PF-127 can improve the survival of embedded HUCMSCs, thereby increasing the repair effect of HUCMSCs on skin wounds. Our results confirmed this conjecture. In the present study, CCK-8 and apoptosis assays demonstrated that 400 μM SAP greatly promoted the survival of HUCMSCs encapsulated in PF-127. Our *in vivo* data revealed that PF-127 plus 400 μM SAP significantly contributed to HUCMSCs-mediated full-thickness wound healing, dermis regeneration, and cell proliferation in mice. These in vivo findings parallel the alterations found in vitro. The current study reveals for the first time that PF-127 and SAP composite improve the efficacy of HUCMSCs-mediated wound healing both in vitro and in vivo. We propose that PF-127 enhances the in-situ residence time of HUCMSCs as well as the antioxidative effect of SAP, which contributes to the great wound healing effect of PF-127/HUCMSCs/SAP hydrogel, while the underlying mechanism remains unclear.

The formation of new blood vessels plays a vital role in orchestrating wound healing. The non-healing nature of chronic wounds is primarily attributable to impaired angiogenesis, which fails to supply adequate nutrients to the injured site^34^. Several lines of evidence suggest that stimulation of angiogenesis is an essential mechanism by which MSCs accelerate wound recovery^34–36^. The proliferation and migration of endothelial cells are considered to be two critical steps in angiogenesis^34^. In vitro experiments, we found that the tube formation and migration of HUVECs were not significantly influenced by PF-127, which demonstrated the biocompatibility of PF-127 hydrogel. Recent studies have shown that stem cells can release a variety of bioactive molecules, such as growth factors, cytokines, chemokines, extracellular matrix (ECM), and small molecules. These bioactive molecules are present in the CM or secretomes, and stem cells use these bioactive molecules to help tissue regeneration^37^. Our results showed that HUCMSC-CM could promote angiogenesis and migration of HUVECs in vitro, suggesting that HUCMSCs may promote the functional recovery of vascular endothelial cells through paracrine effects. This acellular approach has enormous advantages over traditional treatment options and is therefore receiving increasing attention. Furthermore, the pro-angiogenic effect of HUCMSCs is further reinforced by the application of SAP in vitro and in vivo; these results are similar to previous literature reports^38^. Not yet known is the signaling mechanism by which HUCMSCs promote angiogenesis.

VEGF is a definite factor that promotes the permeability of capillaries, the mitogenesis and migration of vascular endothelial cells, matrix degeneration, and vessel tube formation^39^. VEGF alone or in combination with other therapy has been employed to treat chronic wounds^40^. Our study showed that the protein level of VEGF, which almost had no expression in the wounds of PF-127 group, was markedly increased in PF-127/HUCMSCs and PF-127/HUCMSCs/SAP groups, which is coincident with the immunostaining staining results of CD31. Taken together, PF-127/HUCMSCs/SAP hydrogel can efficiently promote the angiogenesis of traumatic skin wounds, and the promoted VEGF level plays a vital role in accelerating angiogenesis, thereby promoting tissue repair.

Taken together, the present study discloses a novel and effective hydrogel scaffold to deliver HUCMSCs to treat the traumatic cutaneous defect. SAP is identified as an effective cell membrane stabilizing agent that dramatically improves the survival of PF-127 encapsulated HUCMSCs via promoting cell proliferation, antibacterial action, and neovascularization, and the up-regulation of VEGF may play a crucial role in PF-127/HUCMSCs/SAP induced wound healing. Our results showed that the PF-127/HUCMSCs/SAP hydrogel showed great biological activity and antibacterial properties and has excellent potential in accelerating wound healing, especially in infected wounds.

## Data Availability

The datasets used and/or analyzed during the current study are available from the corresponding author upon reasonable request.
